# Constitutional *de novo* deletion of the *FBXW7* gene in a patient with focal segmental glomerulosclerosis and multiple primitive tumors

**DOI:** 10.1038/srep15454

**Published:** 2015-10-20

**Authors:** Gaia Roversi, Chiara Picinelli, Ilaria Bestetti, Milena Crippa, Daniela Perotti, Sara Ciceri, Fabiana Saccheri, Paola Collini, Pietro L. Poliani, Serena Catania, Bernard Peissel, Fabio Pagni, Silvia Russo, Paolo Peterlongo, Siranoush Manoukian, Palma Finelli

**Affiliations:** 1 Department of Surgery and Translational Medicine, University of Milano-Bicocca, Monza, Italy; 2 Medical Cytogenetics and Molecular Genetics Lab, IRCCS Istituto Auxologico Italiano, Milan, Italy; 3 Department of Medical Biotechnology and Translational Medicine, Università degli Studi di Milano, Milan, Italy; 4 Molecular Bases of Genetic Risk and Genetic Testing Unit, Department of Preventive and Predictive Medicine, Fondazione IRCCS Istituto Nazionale dei Tumori, Milan, Italy; 5 Medical Genetics Lab, San Gerardo Hospital, Monza, Italy; 6 Soft Tissue and Bone Pathology, Histopathology and Pediatric Pathology Unit, Department of Diagnostic Pathology and Laboratory Medicine, Fondazione IRCCS Istituto Nazionale dei Tumori, Milan, Italy; 7 Pathology Unit, Department of Molecular and Translational Medicine, University of Brescia, Brescia, Italy; 8 Pediatric Unit, Fondazione IRCCS Istituto Nazionale dei Tumori, Milan, Italy; 9 Unit of Medical Genetics, Department of Preventive and Predictive Medicine, Fondazione IRCCS Istituto Nazionale dei Tumori, Milan, Italy; 10 IFOM, Fondazione Istituto FIRC di Oncologia Molecolare, Milan, Italy

## Abstract

Multiple primary malignant neoplasms are rare entities in the clinical setting, but represent an important issue in the clinical management of patients since they could be expression of a genetic predisposition to malignancy. A high resolution genome wide array CGH led us to identify the first case of a *de novo* constitutional deletion confined to the *FBXW7* gene, a well known tumor suppressor, in a patient with a syndromic phenotype characterized by focal segmental glomerulosclerosis and multiple primary early/atypical onset tumors, including Hodgkin’s lymphoma, Wilms tumor and breast cancer. Other genetic defects may be associated with patient’s phenotype. In this light, constitutional mutations at *BRCA1*, *BRCA2*, *TP53*, *PALB2* and *WT1* genes were excluded by performing sequencing and MLPA analysis; similarly, we ruled out constitutional abnormalities at the imprinted 11p15 region by methylation specific -MLPA assay. Our observations sustain the role of *FBXW7* as cancer predisposition gene and expand the spectrum of its possible associated diseases.

FBXW7 (F-box and WD repeat domain-containing 7) is a protein belonging to the SCF (SKP1-CUL1-F-box protein) E3 ligase complex, where it exerts a role of recognition and binding of target proteins for their degradation by the ubiquitin proteasome system (UPS). By UPS, FBXW7 regulates a network of proteins involved in cell division, cell growth and differentiation^1^. Several *in vivo* systems, mainly conditional knockout mouse models, evidenced its importance in tissue development as well as in cancer^2^. Indeed, it is believed that FBXW7 exerts a tumor suppressor (TS) activity by degrading oncogenic proteins, including cyclin E, MYC, JUN and Notch^2^.

Moreover, the evidence that FBXW7 governs cellular apoptosis by ubiquitylation of MCL1, a pro-survival BCL2 family member, explains why altered FBXW7 activity enables cells to evade cell death programme^3^.

In consideration of the high number of targeted proteins, it is not surprising the wide spectrum of tumors harboring *FBXW7* somatic mutations. Among these, mutations were most frequent in cholangiocarcinoma and T-cell acute lymphoblastic leukemia, with a different mutation rate scored in other tumors, possibly reflecting the tissue-specificity of its substrates^1^. However, unlike many TS genes, *FBXW7* does not follow a classic model of tumor suppression, as monoallelic alterations appear to be sufficient for cancer development. The two different “haploinsufficient”^4^ and “just enough”^5^ models have been proposed to explain its functional contribution to tumorigenesis.

FBXW7 is an issue of interest as well in the field of pharmacogenomics; it is in fact implicated in the response to specific chemotherapeutics and to mTOR inhibitors^6^,^7^.

To date only two cases of germline *FBXW7* alterations have been reported in literature, arguing for its involvement in genetic predisposition to Wilms tumor (WT)^8^ and renal cell cancer (RCC)^9^. By identifying an additional case, which shows a peculiar phenotype but also shares some clinical features with the two previous reported patients, we endorse precedent findings and expand the spectrum of *FBXW7* potential related diseases.

## Results

### Clinical description

The patient is the first daughter of two unrelated Italian individuals and at age of 34 was referred to oncological genetic counselling to evaluate the occurrence of a cancer predisposition syndrome. At the time of genetic counselling, her sister and mother were 32 and 60 years old, respectively. No other cases of neoplasia were reported in the family, including in the paternal and maternal grandparents, but no information was available on health status of her father. The patient had a weight of 62 kg (75th percentile), a height of 152 cm (3th percentile), and an occipital frontal circumference of 57,5 cm (>97th percentile). At the age of 17, a nephrotic syndrome due to a primary focal segmental glomerulosclerosis (FSGS) was diagnosed and treated with steroid therapy, cyclophosphamide and cyclosporin. The following year, during the immunosuppressive therapy, an Hodgkin’s lymphoma (nodular sclerosing type) stage IA (for right supraclavear node involvement) was identified and treated with radiotherapy of mediastinal, diaphragmatic and supraclavicular lymph nodes. At the age of 24, the patient underwent ovarian resection for a cystadenoma. At the age of 27, after 8 years of dialysis treatment due to the onset of chronic renal failure, she underwent kidney transplant in the left iliac fossa. A year later, the gallbladder was removed for gallstones and diverticulosis. Immunosuppressive therapy with cyclosporine and sirolimus (a mTOR inhibitor) was continued until the age of 32, when a Stage I WT of the naïve right kidney was diagnosed (triphasic nephroblastoma without anaplasia; at immunocytochemical analysis, WT1 was focally expressed at nuclear level by neoplastic cells) and treated by nephrectomy and adjuvant chemotherapy with actinomycin D and vincristine. At the end of the adjuvant treatment, a mTOR inhibitor (everolimus) was maintained as unique immunosuppressant. At the age of 33, the patient was subjected to prophylactic nephrectomy of the residual left kidney due to the presence in the atrophic parenchyma of multiple perilobar nephrogenic rests (PLNRs) that were immunocytochemically reactive for WT1 at nuclear level. At the age of 34, the patient developed an invasive ductal carcinoma of the right breast (G2, ER and PgR positive, p185 3 + at biopsy), that was treated by neoadjuvant chemotherapy with carboplatin and taxol. No surgery was performed due to the identification of a bone metastases in the right humerus and characterized by HER2 amplification. Herceptin was added to the above mentioned chemotherapeutic agents and after the beginning of radiation therapy for bone metastases, capecitabine replaced carboplatin and taxol.

### Investigations at constitutional level

After informed consent approved from the local Ethical Committee for Diagnostic and Research Purposes was obtained, *TP53*, *BRCA1*, *BRCA2*, *PALB2* and *WT1* genes, which mutations could be possibly consistent with patient’s phenotype, were screened by Sanger sequencing and, to exclude large rearrangements, by Multiplex Ligation-dependent Probe Amplification assay (MLPA). No constitutional deleterious alterations were detected. Similarly, as constitutional defects at 11p15 involving the imprinted domain 1 cause non-syndromic WT^10^, we carried out methylation specific MLPA to determine copy number and methylation status at 11p15. Methylation appeared correctly assessed and microdeletions or duplications within the tested region were excluded.

**Figure 1:**
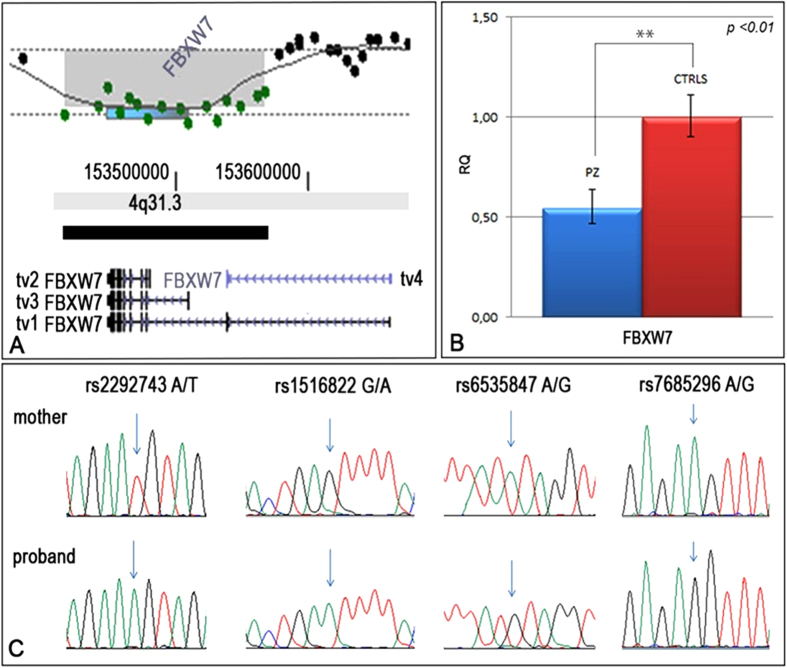
Identification of a *FBXW7* intragenic deletion. (**A**) High-resolution aCGH profile showing the presence of a deletion affetting most of the *FBXW7* gene coding region. (**B**) Relative gene expression analysis of *FBXW7* mRNA in lymphoblastoid cells of the patient (blue) compared to a pull of ten lymphoblastoid cell lines from normal individuals (red), whose value was set to 1. ** = p < 0.01. (**C**) SNP haplotype analysis showed four informative SNPs, located in the deleted genomic region, allowing to establish the deletion’s maternal origin.

Peripheral blood karyotyping revealed a normal female karyotype (46,XX). High resolution aCGH (Array Comparative Genomic Hybridization) 400 K of germline DNA led us to identify a constitutive heterozygous deletion of 157 kb mapping at 4q31.3 (Fig. 1A). The deletion spans between nucleotides 153205202 and 153362047 (UCSC Genome Browser^11^, GRCh37/hg19 assembly), involves the *DEAR* pseudogene and almost the entire *FBXW7* gene, sparing only the first exon of the two transcript variants (tv) at higher molecular weight (Fig. 1A, see tv1 and tv4).

No deletions of similar length were found in the DECIPHER database^12^. However, in this database are reported two *de novo* constitutive heterozygous deletions; the first, which spans 6.9 Mb and involves *FBXW7* and 59 other genes, was found in a 27 years old 46,XX patient showing macrocephaly as the only clinical sign shared with our patient; the second spans 3.7 Mb including 32 genes, and was detected in a 46,XX patient for whom clinical phenotype was not recorded.

Two rare copy number losses affecting the FBXW7 locus, with a size similar to that of the deletion we found, are reported in the Database of Genomic Variant (data obtained from UCSC Genome Browser^11^ GRCh37/hg19 assembly). The first is the merged variant nsv 516218 (6 supporting variants in a sample of 2,026 children mainly Caucasians or African-Americans), which completely spares the tv4 transcript variant; the second is the nsv 523798 (1 supporting variant observed in the same population of 2,026 healthy children), variously affecting all the *FBXW7* isoforms (Supplementary Fig. S1 online).

We studied the normal *FBXW7* allele by PCR and Sanger sequencing and no deleterious mutations were found. Real-time PCR evidenced the decreased levels of *FBXW7* expression in the patient-derived EBV lymphoblastoid cell line as compared to lymphoblastoid cell lines of ten normal controls with a ratio of 0,5:1 (Fig. 1B) that is consistent with the presence of an heterozygous deletion.

To ascertain if the deletion represented a *de novo* copy number variation (CNV) event, a high resolution aCGH analysis was performed on the mother’s constitutional DNA without any evidence of CNV at the *FBXW7* locus. As DNA from the patient’s father was not available, the parental origin of the deleted allele was determined by genotyping the patient’s and her mother’s DNAs at four single nucleotide polymorphisms (SNPs) (rs2292743, rs1516822, rs6535847, rs7685296) mapping inside the deleted region. The haplotypes shown in Fig. 1C indicated the patient received a normal allele from her father, allowing to clarify that the deletion is a *de novo* event affecting the maternal allele.

Aiming at excluding a low level mosaicism in the mother, we designed a PCR assay to specifically amplify only the *FBXW7* deleted allele. Long-range PCR using primers flanking the breakpoints was carried out on both the patient’s and mother’s DNAs. No PCR product was observed after amplification of maternal DNA. The PCR product obtained after amplification of patient’s DNA was sequenced to precisely redefine the breakpoints at nucleotide level (chr4:153197538-AAACAT-153365208, 167.7 kb). Using the patient’s DNA, we prepared serial dilutions mimicking mosaicism and ranging from 50% to 0.1%. PCR product from the deleted allele was observed in each of the DNA dilutions providing evidence, at least in peripheral B lymphocytes, of lack of low level mosaic deletion in the mother.

### Investigations at somatic level

WT tissue was the only available cancer lesion for further molecular analyses. A high resolution CGH array confirmed the known heterozygous *FBXW7* germline deletion, and revealed additional copy number variations. Several small duplications, in the order of few Kbs and involving single genes, a wide duplication of 13 Mb localized in chromosome 20, and two heterozygous deletions of approximately 3 Mb mapping at 1p and 9q were detected. The log2 ratio profile of the X chromosome near -1 was compatible with the loss of the entire chromosome in the tumor (Supplementary Table S1 online). The *FBXW7* locus was investigated by PCR and Sanger sequencing and no deleterious mutations were detected, leading to exclude the presence of a second hit affecting the gene. Real-time PCR showed a decreased level of *FBXW7* expression in WT tissue as compared to a normal renal tissue taken as reference with a ratio of 0,4:1 (Supplementary Fig. S2 online).

**Figure 2:**
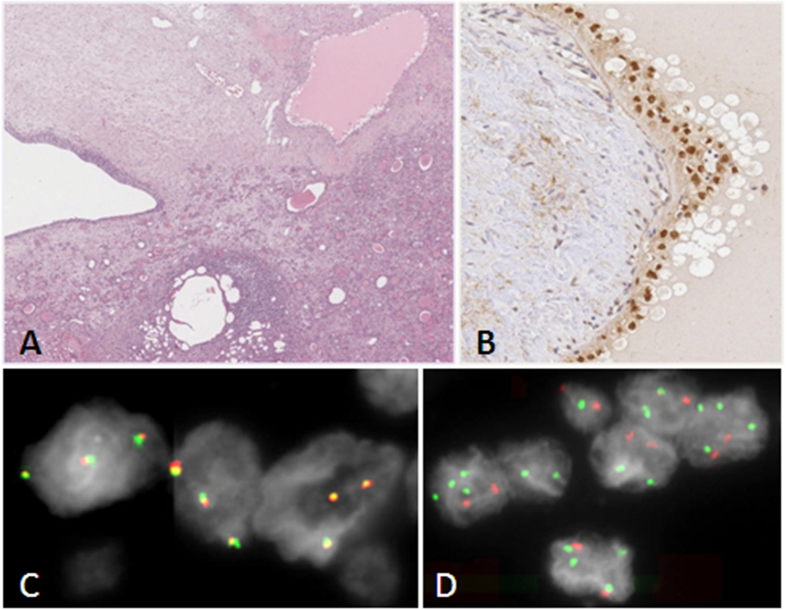
(**A**) Cystic lesions characterized by a cubic or flat epithelium. (**B**) Immunohistochemical analysis for TFE3. (**C**) *TFE3* Break Apart probe FISH on interphase nuclei of the cystic epithelium positive for TFE3 immunostaining. Three cells each with three yellow fused signals, due to lack of traslocation of the 5′ TFE3 red probe from 3′ TFE3 green probe, show each a duplication of a TFE3 allele. (**D**) Dual color FISH with chromosome X (green) and 18 (red) centromeric probes on interphase nuclei of the cystic epithelium positive for TFE3 immunostaining. Most of the cells show two red chromosome 18 centromeric signals and two to four green X chromosome centromeric signals.

Besides PLNRs and marked signs of glomerulosclerosis, the left kidney showed papillary adenomas, microangiomas and cystic cavities, some of which characterized by microcalcifications and lined by cubic or flat cells (Fig. 2A). Strong nuclear immunoreactivity for TFE3 occurred in the cells lining cystic cavities, while the other cells were negative (Fig. 2B). As immunoreactivity for TFE3 is a feature associated with Renal cell Carcinoma (RCC) characterized by the presence of *TFE3* gene fusions^13^, an interphase FISH by using a break apart probe — designed to detect translocations involving the *TFE3* gene at Xp11.2— was performed on formalin-fixed, paraffin-embedded tissues (FFPE) on the area of the cysts immunoreactive for TFE3 of left atrophic kidney. No evidence of a *TFE3* translocation was found; however, a condition of heterogeneity characterized by normal cells mixed with cells with an increased *TFE3* copy numbers (three to five fused signals) was detected (Fig. 2C). A following interphase dual color FISH by using centromeric probes for X and 18 chromosomes displayed that the increased *TFE3* copy number in the immunoreactive cystic epithelium is related to X-chromosome aneuploidy (Fig. 2D). Indeed, while in the surrounding tissue a ratio near 1 between X/18 chromosome signals was scored, inside the cystic lesion the ratio raised to 1,32 (3:2), with a total 35% of nuclei showing 3 or more X chromosome signals and a ratio between X/18 chromosome signals different and major than 1.

## Discussion

Genetic susceptibility to cancer can result from a wide range of constitutive genetic lesions, including rare Copy Number Variations (CNVs) encompassing cancer genes. The search for CNVs in patients with a clinical picture suggestive of an inherited cancer predisposition syndrome can lead to disclose the alteration causative of the cancer susceptibility^14^.

We detected by aCGH the first case of germline deletion confined to the *FBXW7* gene in a patient with a syndromic phenotype characterized by small stature, relative-to-height macrocephaly, focal segmental glomerulosclerosis (FSGS) and multiple, early/atypical onset primitive tumors. The lack of a second hit of *FBXW7* in WT tissue supports evidences for its haploinsufficiency, but *FBXW7* expression levels in WT as compared to a normal kidney showed a ratio (0,4:1) with a slight deviation from the 0,5:1 ratio expected in a clear haploinsufficient status at RNA level. This result can depend on several factors, including a physiological variability of *FBXW7* expression between two RNA specimens, and prevented to clarify the functional contribution of FBXW7 to tumorigenesis, but is in line with the notion that *FBXW7* is not a classic tumor suppressor gene^4^,^5^.

We propose that the identified deletion would have caused increased cancer susceptibility. Although copy number losses affecting only *FBXW7* locus are reported in the DGV, these nsv-s can be classified by frequency as rare (nsv 516218, <1% population frequency) or very rare (nsv 523798, <0,1% population frequency) variants, indeed potentially pathogenetic^14^. The lack of copy number losses in healthy adult population, but not in children, corroborates the hypothesis that the deletion described in our study could predispose to disease in adult life.

The case-patient we studied developed a WT of the right kidney at the age of 32 years old. WT is the most common renal tumor of childhood, occurring rarely in adults, and it can be related to germline mutations causing peculiar genetic syndromes or non-syndromic conditions predisposing to its development^15^. Among the different involved genes^15^, *WT1* germline mutations give rise to isolated WT, isolated forms of FSGS, and to two rare syndromes characterized by the association of male pseudohermaphrodism in 46,XY karyotype with FSGS and gonadoblastoma (Frasier syndrome), or with diffuse mesangial sclerosis and WT (Denys-Drash syndrome), reflecting the complex role of *WT1* in cancer and embryonic urogenital development^16^. The occurrence of diffuse PLNRs in the controlateral kidney represents an additional indication for the presence of a genetic defect conferring a predisposition to cancer. PLNRs, which are abnormally persistent foci of embryonic kidney cells considered to be benign WT precursors, are mainly seen in sporadic tumors and in overgrowth syndromes (idiopathic hemihypertrophy and Beckwith-Wiedemann syndrome), both associated with genetic/epigenetic dysregulation at 11p15 leading to overexpression of IGF-II^17^. Also constitutional defects at 11p15 involving the imprinted domain 1 cause nonsyndromic WT^10^. The patient we studied had a normal 46,XX karyotype and tested negative for *WT1* germline mutations and for 11p15 constitutional abnormalities.

Other known candidate cancer genes, which mutations could be consistent with patient’s phenotype, exist. Specifically, *TP53* is the only one which mutations could be strictly consistent with patient’s phenotype; it should be however noted that the *TP53* associated tumor spectrum generally includes soft-tissue and bone sarcomas. Mutations in *BRCA1, BRCA2*, and *PALB2* could be implicated because of the young age of onset of breast cancer; while *BRCA1* mutations are not associated with WT or Hodgkin’s lymphoma (HL), bi-allelic mutations of *BRCA2* and *PALB2* cause Fanconi Anemia type D1 and Fanconi anemia type N, respectively, of which WT is an associated clinical feature. As no constitutional deleterious mutations were detected in *TP53, BRCA1, BRCA2* and *PALB2*, we excluded all the known genetic syndromes consistent with the overall phenotype.

The involvement of *FBXW7* as a novel WT gene has already been advocated by Williams *et al.* who identified *FBXW7* somatic alterations in about 4% of sporadic WTs^8^. Among the tested cases, the authors also identified one patient carrying a *FBXW7* heterozygous germline mutation (c.45_46insCCT) impairing only the α-isoform of the transcripts, but the coexistence of another germline mutation in *WT1* in the patient hampered any consideration about the contribution of the *FBXW7* alteration to tumor predisposition. Moreover, WT and FSGS might not represent two coincident events, as FBXW7 is a negative regulator of Notch1^2^, and sustained Notch activation is a known mechanism in the pathogenesis of FSGS^18^.

We must however take into account that our patient presented with FSGS closely followed by HL. Although association between nephrotic syndrome and malignancy is known to exist, the specific combination of FSGS and HL is reported as a rare event in literature, and the biological mechanism linking the two diseases has not yet been clarified^19^. We can only speculate that, if we look at FSGS as a paraneoplastic glomerulopathy secondary to an originally hidden HL, we would expect an improvement of the renal condition in parallel with the remission of HL.

With regard to the relationship between *FBXW7* and HL development, less we can infer from the literature; we know of the involvement of *FBXW7* in several forms of acute leukemias^20^, mainly T-cell (T-ALL), as also demonstrated by conditional *FBXW7*–/– knockout models in bone marrow and in T cell lineage developing T-ALL and, thymic lymphoma, respectively^2^. Moreover, a *FBXW7* SNP was demonstrated to act as germline modifier of tumor susceptibility, as it influences the radiation-induced lymphomas development in a p53-dependent manner^21^.

The patient, who developed an early-onset breast cancer, tested negative for mutation screening in the most common breast cancer predisposition genes. The evidences that i) *FBXW7* +/– heterozygous mice show susceptibility to radiation induced tumorigenesis^2^, and that ii) a polymorphism of *FBXW7* may be a risk factor for breast cancer development^22^ let us to include breast cancer in the spectrum of neoplasia related to the *FBXW7* deletion in this patient. Indeed, if it is true that breast cancer is the most common therapy-related subsequent neoplasia after HL, with a clearly demonstrated role for irradiation, it is also true that individual constitutional variations of genes involved in DNA stability (as reported for *FBXW7*^23^) can modulate the risk of second primary cancers after exposure to DNA-damaging agents such as radiations and chemotherapy^24^.

Interestingly, patient’s left kidney showed, besides PLNRs, some cystic lesions characterized by an epithelium reactive for TFE3, which is a marker for a specific RCC subtype, primarily occurring in children and young adults and characterized by Xp11.2 translocations leading to different type of *TFE3* gene fusions^13^. By FISH experiments, we demonstrated that immunoreactivity is probably related to a copy number gain of *TFE3* due to aneuploidy of X chromosome, as yet described in other sporadic cases^25^. This lead us to deduce that *TFE3* is not silenced at renal level by inactivation of additional X chromosomes. It is difficult to explain the significance of this finding, and if these cystic lesions may represent very early cancer lesions, but this observation gains in significance in the light of the case described by Kuiper *et al.* who identified in an adult patient affected by a RCC a familial constitutional translocation t(3;4)(q21;q31) disrupting the *FBXW7* gene at 4q but no genes at 3q^9^.

Unlike our patient, no peculiar clinical features other than a single cancer were described in the two cases studied by Kuiper and Williams, the only ones reporting a *FBXW7* germline alteration. Several reasons may explain this discrepancy: i) differences in the deepening of clinical investigations, ii) types of *FBXW7* molecular defect in the patients, their individual genetic background and variable penetrance of the mutations, and iii) use or combination of different chemotherapeutic treatments based on anti-cancer drugs, or immunosuppressants and inhibitors of mTOR, that is a target of FBXW7^7^.

In conclusion, we extensively characterized the clinical phenotypes of a patient harboring a *de novo* germline deletion in *FBXW7*, expanding the spectrum of signs and diseases possibly associated to this gene. Also because FBXW7 is involved in the response to immunosuppressive^7^ and chemotherapeutic agents^6^, germline *FBXW7* genetic testing should be considered in individuals affected with atypical WT and early RCC where a complex clinical picture exists.

## Methods

### Mutation screening

All the investigations were carried out after that informed consent for Diagnostic and Research Purposes approved by the Ethics Committee of Fondazione IRCCS Istituto Nazionale dei Tumori was obtained. The methods were carried out in accordance with the approved guidelines.

For the detection of constitutional mutations in *TP53*, *BRCA1*, *BRCA2*, *PALB2*, *WT1* and *FBXW7*, genomic DNA was extracted from whole blood using Qiagen DNeasy Blood & Tissue Kit (Qiagen). After amplification, Sanger sequencing of the entire coding region and splice sites of the genes were performed. Moreover, Multiplex Ligation-dependent Probe Amplification assay (SALSA MLPA kit—MRC-Holland) for all the cited genes, with the exception of *FBXW7*, was performed in accordance with the manufacturer’s instructions. For the screening of *FBXW7* somatic mutations, genomic DNA was extracted from FFPE WT by QIAamp DNA FFPE Tissue Kit (Qiagen). Coding exons and splice sites belonging to the four alternative transcripts (tv 1, 2, 3, 4; RefSeq: NM_033632.3, NM_018315.4, NM_001013415.1, NM_001257069.1, respectively) were amplified and sequenced as above described (see Supplementary Table S2 online for primers and conditions).

### MS-MLPA-technique

The MCR-Holland kit, ME-30 RSS_BWS (MRC Holland, Amsterdam, The Netherlands) was used according to the kit instructions. DNAs were extracted from the patient-derived EBV lymphoblastoid cell line, and from normal EBV lymphoblastoid cell lines used as controls. DNAs with and without digestion with the methylation sensitive Hha I enzyme were parallely processed to detect both methylation dysregulation and copy number variation. Data analysis was carried on by the Coffalyzer DB software (Software version: v131211) providing data related to copy number variants and to the methylation status. The latter is defined by the ratio of unmethylated versus methylated DNA, referring each test sample to positive and negative references.

### High-resolution array Comparative Genomic Hybridization (CGH) analysis

Genomic DNA was extracted from whole blood using the GenElute Blood Genomic DNA kit (Sigma-Aldrich, St. Louis, MO) in accordance with the manufacturer’s instructions. Wilm’s tumor DNA was extracted from FFPE sample, based on the method described by van Beers *et al.*^26^ using the Qiagen DNeasy Blood & Tissue Kit (Qiagen). The genome scan was performed using the Human Genome CGH Microarray Kit 400 K for blood, and 244 K for WT (Agilent Technologies, Santa Clara, CA). From both test and normal reference samples, 700 ng of DNA were processed according to the manufacturer’s instructions.

### Quantitative FBXW7 mRNA expression analysis

FBXW7 mRNA expression levels were determined by qPCR on RNA of the patient and ten controls, isolated from lymphoblastoid cells and reverse-transcribed using High-Capacity cDNA Reverse Transcription kit (Applied Biosystems, Foster City, CA). Quantitative real-time RT-PCR, based on the TaqMan methodology, were performed using an ABI PRISM 7700 Sequence Detection System (Applied Biosystems). The amounts of FBXW7 mRNA were calculated using the 2-∆∆Ct method, with glyceraldehyde 3-phosphate dehydrogenase (*GAPDH*) and beta-actin (*ACTB*) as the endogenous-normalizing genes. All assays were provided by Applied Biosystems (TaqMan Gene Expression Assays: ID# Hs00217794_m1 *FBXW7*; Hs99999905_m1 *GAPDH*; Hs99999903_m1 *ACTB*). Real-time data were analysed using the RQ Manager 1.2 software (Applied Biosystems). Statistical analysis was performed by two-tailed Student’s t test and significance was considered at p < 0.01.

The same method was applied to quantify FBXW7 expression levels in WT as compared to a normal kidney. RNAs were obtained from five macrodissected FFPE tissue slice of WT and from a normal FFPE renal specimen by using the RecoverAll™ Total Nucleic Acid Isolation Kit (Life Technologies). A pre amplification of cDNA from FFPE samples was performed prior to real time PCR by using the PreAmp kit (Applied Biosystems). Beta-actin *(ACTB)* and hydroxymethylbilane synthase *(HMBS)* were used as the endogenous-normalizing genes (TaqMan Gene Expression Assays: ID# Hs00609297_m1 HMBS).

### SNP Haplotype Analysis

To establish the parental origin of the deleted allele, sequence-specific PCR was carried out both in patient and patient’s mother. Oligonucleotides and amplification conditions used to amplify four informative SNPs are shown in the Supplementary Table S3 online.

PCRs were performed using the AmpliTaq Gold^®^ kit (Applied Biosystems) and the resulting fragments were sequenced using the Big Dye® Terminator v.3.1 Cycle Sequencing kit (Applied Biosystems). Sequences were then aligned to the human reference genome sequence (human genome GRCh37/hg19 assembly), and analysed with the ChromasPro 1.5 software (Technelysium Pty Ltd., Tewantin QLD, Australia).

### Amplification of the deletion junction fragment and assessment of a possible maternal mosaicism

To localize the deletion breakpoints at nucleotide level in the patient, long-range PCR (LR-PCR) was carried out using the TaKaRa LA Taq^TM^ kit (Takara Bio Inc., Shiga, Japan). The resulting junction fragment was redefine performing sequence-specific PCR using the AmpliTaq Gold^®^ kit (Applied Biosystems). Primer pairs and amplification conditions used to amplify the deletion breakpoint are shown in the Supplementary Table S4 online.

Sequencing was performed as described before. To assess a possible low level maternal mosaicism, the same PCR was performed in the mother. Serial dilutions of the patient’s DNA with a reference DNA corresponding to a mosaicism of the deletion of 50%, 25%, 12.5%, 5%, 2.5%, 1%, 0.5%, 0.25%, 0.1% were carried out to evaluate the PCR sensibility.

### Immunohistochemistry for TFE3 detection

For immunocytochemical studies, 5 μm-thick sections were processed in an automated Ventana BenchMark IHC/ISH instrument using XT ultraview DAB v3 protocols (Ventana System, Tucson, AZ). A rabbit monoclonal anti-TFE3 antibody (MRQ-37, Cell Marque) was applied.

### Interphase FISH for TFE3 detection

FISH analysis was performed on 4 μm FFPE tissue sections using TFE3 (Xp11.2) Break Apart probe (Poseidon) and CEP18, X, Y-alpha satellite probe (Vysis).

Sections were baked o/n at 60 °C, deparaffinized by soaking slides in xylene and then rehydrated. They were heated in pre-treatment solution at 95 °C for 20 minutes, digested with pepsin at 37 °C for 40 minutes and dehydrated. Finally samples were denatured according to the probe manufacturer’s instructions, incubated o/n at 37 °C in a humidified chamber, washed in post-hybridization buffers and counterstained with DAPI/Antifade.

## Additional Information

**How to cite this article**: Roversi, G. *et al.* Constitutional *de novo* deletion of the *FBXW7* gene in a patient with focal segmental glomerulosclerosis and multiple primitive tumors. *Sci. Rep.* **5**, 15454; doi: 10.1038/srep15454 (2015).

## Supplementary Material

Supplementary Information
