# Supplementary material for: Constitutional de novo deletion of the FBXW7 gene in a patient with focal segmental glomerulosclerosis and multiple primitive tumors
Source: Sci Rep. 2015 Oct 20;5:15454. doi: 10.1038/srep15454 (PMC4612309; doi:10.1038/srep15454)
Supplement: Supplementary Information [file srep15454-s1.pdf]

SUPPLEMENTARY INFORMATION

CONSTITUTIONAL DE NOVO DELETION OF THE *FBXW7* GENE IN A PATIENT WITH FOCAL SEGMENTAL GLOMERULOSCLEROSIS AND MULTIPLE PRIMITIVE TUMORS

Gaia Roversi[1], Chiara Picinelli[2], Ilaria Bestetti[2-3], Milena Crippa[2], Daniela Perotti[4], Sara Ciceri[4], Fabiana Saccheri[5], Paola Collini[6], Pietro L.Poliani[7], Serena Catania[8], Bernard Peissel[9], Fabio Pagni[1], Silvia Russo[2], Paolo Peterlongo[10], Siranoush Manoukian[9*], Palma Finelli[2-3]

[1]Department of Surgery and Translational Medicine, University of Milano-Bicocca, Monza, Italy
[2]Medical Cytogenetics and Molecular Genetics Lab, IRCCS Istituto Auxologico Italiano, Milan, Italy
[3]Department of Medical Biotechnology and Translational Medicine, Università degli Studi di Milano, Milan, Italy
[4]Molecular Bases of Genetic Risk and Genetic Testing Unit, Department of Preventive and Predictive Medicine, Fondazione IRCCS Istituto Nazionale dei Tumori, Milan, Italy
[5]Medical Genetics Lab, San Gerardo Hospital, Monza, Italy
[6]Soft Tissue and Bone Pathology, Histopathology and Pediatric Pathology Unit, Department of Diagnostic Pathology and Laboratory Medicine, Fondazione IRCCS Istituto Nazionale dei Tumori, Milan, Italy
[7]Pathology Unit, Department of Molecular and Translational Medicine, University of Brescia, Brescia, Italy
[8]Pediatric Unit, Fondazione IRCCS Istituto Nazionale dei Tumori, Milan, Italy
[9]Unit of Medical Genetics, Department of Preventive and Predictive Medicine, Fondazione IRCCS Istituto Nazionale dei Tumori, Milan, Italy
[10]IFOM, Fondazione Istituto FIRC di Oncologia Molecolare, Milan, Italy

[*]Correspondence to: Siranoush Manoukian, Unit of Medical Genetics, Department of Preventive and Predictive Medicine, Fondazione IRCCS Istituto Nazionale dei Tumori, via Venezian 1, 20133, Milan, Italy; Siranoush.Manoukian@istitutotumori.mi.it , +39-02-23902809

SUPPLEMENTARY FIGURE S1

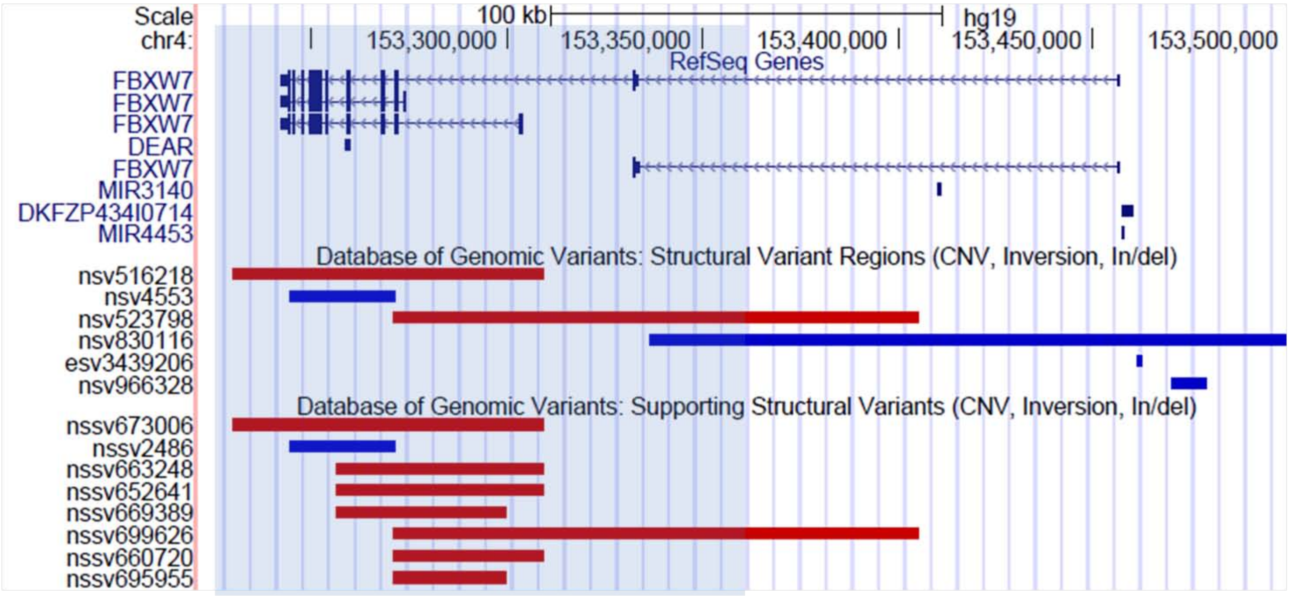

Supplementary Figure 1. Screenshots of the Copy Number Variations affecting the FBXW7 locus reported in the DGV database (obtained from UCSC Genome Browser[11], GRCh37/hg19 assembly) in relation to the width of the identified deletion (light blue area); Red bars: losses; Blue bars: gains; nsv: NCBI Structural Variant (representative variant that merges more supporting variants); nssv: NCBI Supporting Structural Variant (variant called in a single sample/individual).

SUPPLEMENTARY FIGURE S2

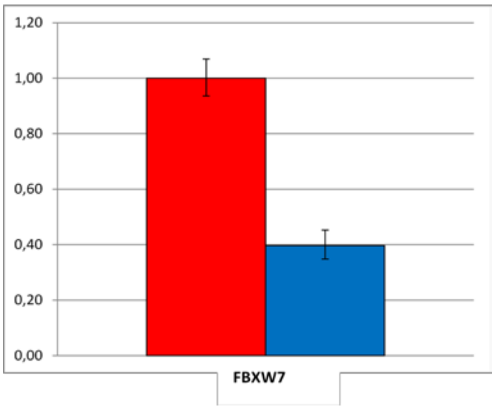

Supplementary Figure 2. Relative gene expression analysis of *FBXW7* mRNA in Wilms tumor as (blue) compared to a normal renal tissue (red), whose value was set to 1. ($p<0.01$)

SUPPLEMENTARY TABLE S1

Results of high resolution array CGH of Wilms tumor tissue

Chromosome and Cytoband	Aberration type and width	Genes#
1p36.32-p36.31	Del 3.3 Mb	Several, among which TP73, AJAP1, DFFB
3p21.31	Del 90 kb	DOCK3
4q12	Dup 111 kb	POLR2B* , IGFBP7
4q31.3	Del 151 kb	FBW7*
6p22.1	Dup 213 kb	histone gene cluster (HIST1 H1A-H3E), HFE
7p15.2	Dup 125 kb	homeobox genes cluster (HOXA1-13)
8q21.11	Dup 224 kb	RDH10*, STAU2*
8q24.21	Dup 166 kb	ASAP1*
9q33.1	Del 3.5 Mb	ASTN2*,TLR4, BRINP1
11q21	Dup 243 kb	MAML2*
13q21.33	Dup 354 kb	DACH1*
13q32.2	Dup 113 kb	FARP1
14q24.1	Dup 976 kb	RAD51B*, ZFP36L1, ACTN1*
17p13.1	Dup 71 kb	VAMP2, TMEM107, MIR4521, C17orf59, AURKB, LINC00324
20q13.12-q13.33	Dup 13 Mb	Several, among which AURKA
X	Del	All, among which WTX

# only genes possibly related to cancer are reported for aberrations of Mbases
* interrupted genes by the aberration

SUPPLEMENTARY TABLE S2

Primers used for *FBXW7* amplification and sequencing of DNA extracted from Wilms Tumor

SNPs	***Designation***	***Primer sequence (5'-> 3')***	***Annealing T(°C)***	***PCR size (bp)***
1A-1	*Forward*<br>*Reverse*	AGCTGGCTTTTGGAAATGAA<br>CTGTCCTTGCTGGGAATCAT	56	243
1A-2	*Forward*<br>*Reverse*	ATGATTCCCAGCAAGGACAG<br>CGGGCAGGTCCACAATACTA	56	252
1A-3	*Forward*<br>*Reverse*	CCCGTTCACCAACTCTCCT<br>CATTTGTACTCAGATTGTCCCATT	56	249
1B	*Forward*<br>*Reverse*	CCTTGGGTTCTCAGCCTTTT<br>AAAAGAGGCCAAGGTACTTCA	56	335
1C-1	*Forward*<br>*Reverse*	CAGGGCATAGTCTCCTCCAA<br>AGCAGAACCGGCAACAAAAC	54	156
1C-2	*Forward*<br>*Reverse*	GTTTTGTTGCCGGTTCTGCT<br>TCTCAGGCAGGCATACACAC	54	264
2	*Forward*<br>*Reverse*	CCATGCTGACTCAAGATTTGATA<br>TTCCGGTAATCTCAAAATGTGTT	56	288
3	*Forward*<br>*Reverse*	TGCCAGATCATCATTCTTTG<br>GCAGCAATTAAGTGAGGCATT	56	300
4	*Forward*<br>*Reverse*	GCCTGTAATTTGGGACATCTG<br>TGTTTAAAGGTGGTAGCTGTTGA	56	264
5	*Forward*<br>*Reverse*	TCAAGTATCTCATCCTGTGGAGAA<br>TGTTTTCAGAATCACTCTGCTTTT	56	281
6	*Forward*<br>*Reverse*	TGGTTTTGAGCAGAGAGATGG<br>ACAGTTTGCCAAGTGAAATAGT	54	326
7	*Forward*<br>*Reverse*	GCCTTCATTTTTCTCTTCACCA<br>CCAGTTGCTACTTGCAATGAT	54	262
8	*Forward*<br>*Reverse*	TCACTTTTCCTTTCTACCCAAAA<br>AGGGCCCAAATTCACCAATA	52	291
9	*Forward*<br>*Reverse*	TTAAAAATTCTAAACGTGGGTTTT<br>GGAGAGCATTTAAGGGAGAGA	52	321
10-A	*Forward*<br>*Reverse*	TCCTGGCATTACCTGTTTCC<br>ATGCAATTCCCTGTCTCCAC	56	189
10-B	*Forward*<br>*Reverse*	TGTTTGGGATGTGGAGACAG<br>AACCATTCTGTATGAGGTTGACTC	54	271
11	*Forward*<br>*Reverse*	TGAGGACATGGGTTTCTAAATATG<br>GAAGGGCAGGGAGTATATCG	58	467

SUPPLEMENTARY TABLE S3

Primers used for SNPs haplotype analysis

SNPs	*Designation*	*Primer sequence (5'-> 3')*	*Annealing T(°C)*	*PCR size (bp)*
rs2292743	*Forward*<br>*Reverse*	TTACCTCAGCATTTCCCAAA<br>CACATTCTGCAGGGGAAAAT	54,2	430
rs1516822	*Forward*<br>*Reverse*	CAAACCCTGTGGACAGCTAGA<br>AACTGAGCCCTCTGTCCTTTC	59,6	579
rs6535847	*Forward*<br>*Reverse*	GCAAGTCTGGGAAAATATTGG<br>GCAAGGTTTTGTCATGTAGGG	56,7	639
rs7685296	*Forward*<br>*Reverse*	TCTGTCAGGGAAGCATTTAGG<br>TTGGGTTCCAGGAATGAAAG	56,5	426

SUPPLEMENTARY TABLE S4

Primers used for amplification of the deletion junction fragment

	Designation	*Primer sequence (5'->3')*	*Annealing T(°C)*	*PCR size (bp)*
**LR-PCR**	*Forward*<br>*Reverse*	AGAAAAGAAATGCTGCATCAATACAGTG<br>GTGAGTGTAGAGATGAAGCCAAATTTCA	60,7	2290
**PCR**	*Forward*<br>*Reverse*	GCATCTAGAGATCAGGTCAGAAGTGGAT<br>TCCTGTTTTACCTTGTTTCTATGCCTTG	62,9	1252
